# Effects of an mHealth Brisk Walking Intervention on Increasing Physical Activity in Older People With Cognitive Frailty: Pilot Randomized Controlled Trial

**DOI:** 10.2196/16596

**Published:** 2020-07-31

**Authors:** Rick YC Kwan, Deborah Lee, Paul H Lee, Mimi Tse, Daphne SK Cheung, Ladda Thiamwong, Kup-Sze Choi

**Affiliations:** 1 Centre for Gerontological Nursing, School of Nursing The Hong Kong Polytechnic University Hong Kong Hong Kong (China); 2 School of Nursing The Hong Kong Polytechnic University Hung Hom Hong Kong (China); 3 College of Nursing University of Central Florida Orlando, FL United States

**Keywords:** cognitive frailty, brisk walking, smartphone, moderate-to-vigorous physical activity, older people

## Abstract

**Background:**

Cognitive frailty is the coexistence of physical frailty and cognitive impairment and is an at-risk state for many adverse health outcomes. Moderate-to-vigorous physical activity (MVPA) is protective against the progression of cognitive frailty. Physical inactivity is common in older people, and brisk walking is a feasible form of physical activity that can enhance their MVPA. Mobile health (mHealth) employing persuasive technology has been successful in increasing the levels of physical activity in older people. However, its feasibility and effects on older people with cognitive frailty are unclear.

**Objective:**

We aimed to identify the issues related to the feasibility of an mHealth intervention and the trial (ie, recruitment, retention, participation, and compliance) and to examine the effects of the intervention on cognitive function, physical frailty, walking time, and MVPA.

**Methods:**

An open-label, parallel design, randomized controlled trial (RCT) was employed. The eligibility criteria for the participants were age ≥60 years, having cognitive frailty, and having physical inactivity. In the intervention group, participants received both conventional behavior change intervention and mHealth (ie, smartphone-assisted program using Samsung Health and WhatsApp) interventions. In the control group, participants received conventional behavior change intervention only. The outcomes included cognitive function, frailty, walking time, and MVPA. Permuted block randomization in 1:1 ratio was used. The feasibility issue was described in terms of participant recruitment, retention, participation, and compliance. Wilcoxon signed-rank test was used to test the within-group effects in both groups separately.

**Results:**

We recruited 99 participants; 33 eligible participants were randomized into either the intervention group (n=16) or the control (n=17) group. The median age was 71.0 years (IQR 9.0) and the majority of them were females (28/33, 85%). The recruitment rate was 33% (33/99), the participant retention rate was 91% (30/33), and the attendance rate of all the face-to-face sessions was 100% (33/33). The majority of the smartphone messages were read by the participants within 30 minutes (91/216, 42.1%). ActiGraph (58/66 days, 88%) and smartphone (54/56 days, 97%) wearing compliances were good. After the interventions, cognitive function improvement was significant in both the intervention (*P*=.003) and the control (*P*=.009) groups. The increase in frailty reduction (*P*=.005), walking time (*P*=.03), step count (*P*=.02), brisk walking time (*P*=.009), peak cadence (*P*=.003), and MVPA time (*P*=.02) were significant only in the intervention group.

**Conclusions:**

Our mHealth intervention is feasible for implementation in older people with cognitive impairment and is effective at enhancing compliance with the brisk walking training program delivered by the conventional behavior change interventions. We provide preliminary evidence that this mHealth intervention can increase MVPA time to an extent sufficient to yield clinical benefits (ie, reduction in cognitive frailty). A full-powered and assessor-blinded RCT should be employed in the future to warrant these effects.

**Trial Registration:**

HKU Clinical Trials Registry HKUCTR-2283; http://www.hkuctr.com/Study/Show/31df4708944944bd99e730d839db4756

## Introduction

Cognitive frailty is a heterogeneous clinical syndrome characterized by the coexistence of both physical frailty and cognitive impairment without being severe enough to fulfil the criteria for dementia [1]. Physical frailty is a phenotype of slowness, muscle weakness, less physical activity, exhaustion, and weight loss [2]. Cognitive frailty is common in community-dwelling older people, with a prevalence of 2.4%-8.9% [3,4]. It is an at-risk state for many adverse health outcomes, including dementia, dependency, and mortality [5-7].

Physical activity is protective against the progression of cognitive frailty because it optimizes the neurobiological conditions that cause cognitive frailty (eg, glucose metabolism, sarcopenia, insulin resistance) [8-10]. Physical inactivity is defined by the World Health Organization (WHO) as the performance of less than 150 minutes of moderate-to-vigorous physical activity (MVPA) per week [11]. The WHO has also advised that engaging in MVPA for 150 minutes per week, with each session lasting not less than 10 minutes, will lead to health benefits [11]. Physical inactivity is one of the key phenotypical characteristics of frailty [2]. There is evidence that the intensity of physical activity plays an important role in yielding favorable health outcomes in older people. A study showed that replacing sitting time with MVPA was associated with a significant decrease in frailty but replacing sitting time with light physical activity was not [12]. Despite the beneficial effects of MVPA, physical inactivity is still common in older people, with a prevalence of over 60% in the United States and over 40% in China [13,14]. A systematic review showed that the prevalence of physical inactivity increases with age in older people [15].

Walking is the most common and inexpensive form of physical activity for older people and makes up approximately 80% of the total amount of physical activity that they engage in during their leisure time [16]. An ActiGraph accelerometer validation study showed that walking at a speed of >3.2 km/h or >100 steps/min fulfilled the criteria of MVPA, which is 3 metabolic equivalents in older people [17,18]. Therefore, brisk walking is a feasible form of physical activity for older people to enhance their MVPA.

Behavioral change interventions employing various behavioral change techniques have been successful at changing the behavior of different populations, from being physically inactive to becoming physically active [19]. In the process of ageing, many factors hinder older people from leading a physically active life, including poor health, a lack of company, a lack of interest, a lack of skills, and a lack of opportunities [20,21]. These factors can diminish the effects of a behavioral change intervention aimed at increasing their levels of physical activity. A systematic review showed that the effect size of a behavioral change intervention in older people was small (*d*=0.14) because many self-regulation intervention techniques that are effective for younger adults may not be effective for older adults [22]. There is a need to maximize the effect for this vulnerable group to ensure that older people with cognitive frailty are physically active enough to promote their health.

Mobile health (mHealth, also known as eHealth) refers to the health services delivered or enhanced through mobile/electronic-related technology [23]. Persuasive technology is a branch of mHealth in which the aim is to use digital technology to guide users to change their attitudes and behavior by enhancing the effects of behavioral change techniques [24]. mHealth interventions show promise in encouraging older people to increase their levels of physical activity as reported in systematic reviews [25-28]. Because there is a lack of properly designed trials in this area, none of these systematic reviews drew conclusions on whether the mHealth is more effective or can enhance the effect of the conventional intervention to promote physical activity in older people.

Older people with cognitive frailty are more vulnerable than robust older people to engage in physical activity because cognitive impairment and physical frailty reduce their intrinsic motivation through mechanisms such as neurological and muscular damage [29,30]. They have much lower baseline motivation than the robust older people. Baseline motivation is known to be associated with the effect of behavioral changes [31]. Moreover, compared to the robust people, older people with cognitive frailty have a lower baseline physical activity level [2]. The effect of a behavioral intervention promoting physical activity is known to be weaker in people with lower baseline physical activity [32]. Thus, the promising effect of eHealth interventions in robust older people may be hindered in translating to older people with cognitive frailty, let alone their effects on the clinical outcomes of cognitive frailty.

In the previous trials, most of the mHealth interventions that were included in the reviews used only websites, DVDs, and texting instead of face-to-face physical activity training and health education [25-28]. Recently, studies have shown that the newly developed technologies, including wearable devices and social media, showed promising effects in promoting physical activity in many populations (eg, young people, cancer survivors) [33-35]. However, there is a lack of trials examining whether the new mHealth technologies are more effective than the conventional behavior change programs (eg, face-to-face behavioral counselling, education). We hypothesize that the mHealth intervention employing the recently developed technologies is feasible and can enhance the behavioral change effect to promote physical activity in older people with cognitive frailty and eventually lead to favorable clinical outcomes (ie, amelioration of cognitive frailty). The aims of this study were to identify issues relating to the feasibility of the interventions and the trial (ie, recruitment, retention, participation, and compliance) and to examine the preliminary effects of the intervention by testing the following hypothesis: (1) the mHealth intervention significantly increases cognitive function, reduces physical frailty, increases walking behaviors, and increases MVPA and (2) the conventional behavior change intervention does not significantly increase cognitive function, reduce physical frailty, increase walking behaviors, or increase MVPA.

## Methods

### Trial Design

An open-label, parallel design (1:1 ratio), randomized controlled trial (RCT) was employed. This pilot trial has been registered with the Hong Kong University Clinical Trials Registry (HKUCTR-2283). This section reports the methods employed in the trial by following the CONSORT-EHEALTH checklist (Multimedia Appendix 1) [36].

### Settings

This study was conducted in community settings. The participants were recruited from 2 elderly community centers in Hong Kong during the period of May 2018 to June 2019. Elderly community centers regularly provide recreational and social activities for community-dwelling people above the age of 60 years.

### Participants

The inclusion criteria for the participants were age ≥60 years and having cognitive frailty, which was operationally defined as the coexistence of mild cognitive impairment (MCI) and physical frailty (including both frailty and prefrailty) [6]. MCI was confirmed by the following criteria put forward by the National Institute on Aging-Alzheimer’s Association [37]: (1) self-reported or informant-reported cognitive complaints, (2) objective cognitive impairment, as defined by a Clinical Dementia Rating of 0.5 and a Montreal Cognitive Assessment (MoCA) score of <25 [38,39], (3) preservation of one’s independence, as defined by the Lawton’s Instrumental Activity of Daily Living score of >14 [40], and (4) no diagnosed dementia, as observed in the medical record, as defined by the Diagnostic and Statistical Manual of Mental Disorders, Fifth Edition criteria for Major Neurocognitive Disorders [41]. Physical frailty from being prefrail to frail was defined by a Fried Frailty Index (FFI) score of 1-5 [2]. Physical inactivity was operationally defined by MVPA<150 min in the last 7 days. It was measured by structured interviews guided by a list of MVPAs with reference to the Physical Activity Scale for the Elderly [42]. The exclusion criteria for the participants were having impaired mobility because older people cannot briskly walk outdoors, as defined by the modified Functional Ambulatory Classification score of <7 [43], depressive symptomatology because they have poor motivation as defined by a Geriatric Depression Scale score of ≥8 [44], or probable dementia (ie, MoCA<20 or clinical dementia rating ≥1) [45] because the underdiagnosis of dementia is common, implying that some cases of dementia do not appear on medical records [46].

### Intervention Group and Control Group Interventions

As shown in Table 1, in the intervention group, both the conventional behavior change intervention and the mHealth intervention will be implemented. In the control group, only the conventional behavior change intervention will be implemented.

**Table 1:** Intervention groups and intervention procedures.

Groups and intervention procedures		Conventional behavior change intervention		mHealth intervention	
**Intervention group**					
	Reducing the difficulty of changing the target behavior by setting short-term goals		Face-to-face meetings		WhatsApp
	Personalization of goals		Face-to-face meetings		WhatsApp + Samsung Health
	Messages of praise		Face-to-face meetings		WhatsApp + Samsung Health
	e-reminders		N/A^a^		WhatsApp + Samsung Health
	Use of validity tested-devices		N/A		Samsung Galaxy smartphone
	Integration of self-tracking		N/A		Samsung Health
	e-coaching		N/A		WhatsApp + Samsung Health
**Control group**					
	Brief activity counselling		Face-to-face meetings		N/A
	Telephone follow-up		Telephone		N/A
	Health education		Face-to-face meetings		N/A
	Exercise training (brisk walking)		Face-to-face meetings		N/A

^a^Not applicable.

#### Conventional Behavior Change Intervention

Conventional behavior change interventions have been shown to have a significant effect on prompting physically inactive older people to increase their levels of physical activity by employing various measures [22]. A large-scale study (N=878) showed that a behavioral change intervention that included brief activity counselling using motivational interviewing, regular telephone and face-to-face support, health education, and exercise training significantly increased the physical activity of the older people [47]. As shown in Table 1, these 4 components were adopted in this study to formulate the conventional behavior change intervention. To materialize these components, the procedures in a large-scale study were adopted for the *motivational interviewing* and *regular telephone support* components [47]. For the *health education* component, the educational resources for cognitive frailty from both the National Health Service and the Alzheimer’s Association were utilized because they provide internationally reliable health education information that is comprehensible to laypeople [48,49]. The *exercise training* (ie, training in brisk walking) component adopted the contents of the brisk walking training program devised by the Hong Kong Leisure and Cultural Services Department [50].

#### mHealth Intervention

A systematic review of 32 publications indicated that mHealth interventions are effective when they include (1) reduction in the difficulty of the target behaviors to be changed, (2) personalization of goals, (3) messages of praise, (4) e-reminders, (5) use of validity-tested devices, (6) integration of self-tracking, and (7) e-coaching [51]. As shown in Table 1, these 7 components were adopted in this study to formulate the mHealth intervention.

To materialize these components, we adopted a simple target behavior (ie, brisk walking) that reduces the difficulty of the *target behavior to be changed*. The Samsung Galaxy smartphone J2 with 2 apps (ie, Samsung Health and WhatsApp) was used. This smartphone was chosen because it has many accurate sensors (eg, a triaxial accelerometer) and is a *validity-tested device* that can be used to accurately measure step counts and walking velocity when placed in a pant pocket or a backpack during free walking and has a mean absolute percentage error of less than 3 [52]. Samsung Health is a physical activity autotracking app. It autonomously and continually monitors the walking behaviors (eg, steps, walking speed, walking time, physical activity intensity) of the users. It also coaches users to set individualized goals, logs all the physical activity data, provides immediate onsite rewards and performance reviews, suggests tailored walking, provides real-time feedback, and allows comparisons to be made with peer participants. Samsung Health is used because it allows *e-reminders* to be sent to the users to remind them to perform their walking exercise and provides *self-tracking* of the walking behaviors and the amounts of physical activity with immediate feedback at the scene. WhatsApp is a communication app that allows users to send text messages and voice messages, to make voice calls and video calls, and to share images, documents, user locations, and other media. WhatsApp was used because it allows *e-coaching, personalization of goal settings*, *and messages of praise* to be remotely provided by the research assistants.

#### Implementation Procedures

A trained interventionist provided the interventions. One interventionist was trained to deliver the interventions to all the participants because we wanted to minimize the inter-interventionist variance during the stage of the pilot trial. The interventionist was a baccalaureate graduate with a major in psychology who had received theoretical training related to behavioral change. Before the implementation of the interventions, the interventionist completed training in brisk walking provided by a nursing academic specializing in gerontology.

As shown in Table 2, the total intervention period lasted for 12 weeks. In both the groups, health education was launched in week 1. Exercise training (ie, training in brisk walking) was conducted in weeks 1 and 2. One training session was conducted in an elderly center and another in a real practice environment close to the participants (ie, a public park). Face-to-face meetings were conducted 3 times in weeks 4, 8, and 12. In the control group, in addition to the above, 2 telephone follow-up meetings were conducted in weeks 6 and 10. In the intervention group, the follow-up sessions were conducted using a smartphone with WhatsApp and Samsung Health, instead of with a telephone, from weeks 3 to 12, immediately after the participants received training on how to use the smartphone. Messages (eg, praise messages, e-reminders, personalized goals, coaching) were sent to the participants at least once a week.

**Table 2:** Intervention implementation schedule.

Groups and interventions		Weeks											
		1	2	3	4	5	6	7	8	9	10	11	12
**Intervention group**													
	mHealth^a^	N/A^b^	N/A	SF^c^	SF	SF	SF	SF	SF	SF	SF	SF	SF
	CBC^d^	ET1^e^ and HE^f^	ET2	ST^g^	F2F^h^	N/A	N/A	N/A	F2F	N/A	N/A	N/A	F2F
**Control group**													
	CBC	ET1 and HE	ET2	N/A	F2F	N/A	TF^i^	N/A	F2F	N/A	TF	N/A	F2F

^a^mHealth: mobile health.

^b^Not applicable.

^c^SF: smartphone follow-up (WhatsApp + Samsung Health)

^d^CBC: conventional behavior change.

^e^ET: exercise training.

^f^HE: health education.

^g^ST: smartphone training.

^h^F2F: face-to-face meeting.

^i^TF: telephone follow-up.

Tailored dosages were employed. Tailoring refers to the adjustment of intervention implementation strategies to address varying levels of the barrier to changing particular behaviors. Tailoring is needed because these barriers affect the effectiveness of the behavioral change [53]. The varying barrier in this population is mainly the level of the physical fitness at baseline. The training target was adjusted to the baseline physical fitness. The latest guideline recommends that older people should be encouraged to increase their level of activity by small amounts, rather than focusing on immediately reaching the recommended level [54].

As shown in Table 3 and Table 4, the intervention was tailored to 2 aspects: (1) setting personalized goals and (2) contacting participants. The personalized physical activity goals were set according to 4 principles: (1) practice availability, (2) baseline fitness, (3) previous performance, and (4) personal wish. WhatsApp messages were sent to the participants in response to 3 triggers: (1) weekly routine messages, (2) when there was no brisk walking for more than 2 days, and (3) when the weekly goal was achieved earlier than expected. The details of the tailoring procedures are shown in Multimedia Appendix 2.

Therefore, the weekly training goals (eg, relating to walking speed, walking time, number of steps) were tailored according to the participants’ individual level of physical fitness at baseline and to their progress. The training goal at the first level was to increase the number of MVPA minutes, while the goal at the second level was to increase the number of MVPA minutes to above 150 min/week at a rate agreed upon by both the participants and the research assistants.

**Table 3:** Description of the tailored strategies as per personalized goals in the mHealth intervention.

Principles	Actions
Practice availability	Set number of time slots and set available periods to practice brisk walking
Baseline fitness	Prefrail: start with a goal of 5-10 sessions/week^*^, Frail: start with a goal of 3-5 sessions/week^*^
Previous performance	Achievers: add 2-5 minutes/session and add 1 session/week; Nonachievers: goal remains as the best performance the participant has ever achieved in the previous weeks
Personal wish	Compromised with the participants according to their wishes (eg, confidence, motivation)

*A session is preset as a 10-minute brisk walking session.

**Table 4:** Description of the tailored strategies by contacting the participants in the mHealth intervention.

Triggers	Contents
Weekly routine messages	A set of messages on the health benefits of brisk walking and a summary of the participants’ performance in the previous week
When there is no brisk walking for more than 2 days	e-reminders and e-coaching
When the weekly goal is achieved before the end of the week	Messages of praise

#### Outcomes

Trained research assistants collected the data at the baseline, which is the week before the randomization and commencement of the intervention (T0) and at 1 week after the completion of the intervention (T1). The demographic and clinical characteristics were measured at baseline. The outcome variables were collected at both T0 and T1. The 4 outcomes were as follows:

1. Cognitive function was measured using the MoCA [38]. MoCA contains 30 dichotomous items. The correct answer on 1 item will be accorded a score of 1 point. Total scores range from 0 to 30, with a higher score indicating better cognitive function. The test has been found to have good validity in detecting MCI (sensitivity 0.90, specificity 1.00) [38].
2. Frailty was measured using the FFI [2], which quantifies the phenotypes of frailty according to 5 components (ie, weight loss, exhaustion, low physical activity, slow gait, and weakness) by using physical performance tests and questionnaires following Fried’s guideline. FFI scores range from 0 to 5, with 1 point assigned for the presence of 1 component. A higher FFI score indicates a higher frailty level. Those with 0, 1-2, or 3-5 point(s) are classified respectively as robust, prefrail, or frail, respectively.
3. Walking was measured using a wrist-worn ActiGraph GT3X+, which is a valid step counter because it can estimate 97.5% of the observed steps in a free-living environment [55]. Walking was quantified according to the total walking time, brisk walking time (>100 steps/min), step counts, and 1-minute peak cadence. The participants were instructed to wear the ActiGraph during the data collection time intervals (ie, T0 and T1) for 24 hours a day and for 7 days. They were allowed to remove the ActiGraph only on special occasions (eg, bathing) that were expected to amount to less than 1 hour per day. Although Samsung Health is valid for counting steps, its data could not be extracted into minute-by-minute units for a precise data analysis. An ActiGraph was therefore used to measure the amount of walking that the participants engage in.
4. Physical activity was also measured using a wrist-worn ActiGraph GT3X+ because it has a good criterion validity for differentiating MVPA from non-MVPA compared to indirect calorimetry (cutoff 6,367, sensitivity 0.70, specificity 0.87, area under the curve 0.83; *P*<.001) in older people when walking on a treadmill at different speeds [56]. An MVPA minute is defined as a minute in which the ActiGraph has recorded physical movements (ie, vector magnitude) of above 6367 counts/min [56]. Only 10 minutes of continuous MVPA minutes were counted as valid MVPA minutes because only sessions of more than 10 minutes of continuous MVPA are considered beneficial, as recommended by the WHO [11]. Physical activity is quantified by valid MVPA minutes measured in 7 consecutive days because a 7-day interval is adequate for understanding the pattern of the routine physical activity engaged in by an individual and is widely used as a standard in studies on physical activity [55,57]. Only MVPA minutes measured on valid days count as valid MVPA minutes (ie, ActiGraph wearing time >10 hours/day) for a valid period (ie, valid days >3 days) [58,59].

### Sample Size

There are no previous studies reporting the effects of an eHealth intervention promoting physical activity specifically in older people with cognitive frailty. In a systematic review, eHealth interventions were found to increase MVPA for 8.6-16.0 min/day in the general older population [28]. A study showed that the older people with MCI generally performed MVPA for 24.1 (SD 18.7) min/day [60]. Considering that the effect on older people with MCI is lower because of their lower motivation [29], we adopted a conservative approach to estimate a between-group difference of 8.6 MVPA min/day with a baseline MVPA time of 24.1 (SD 18.7) min/day (ie, *d*=0.46).

This is a pilot trial, which was not aimed to test the effect by a full-powered study. Instead, this pilot trial aimed to estimate the preliminary effect in a small sample to provide a reference of effect for the main trials to estimate the sample size. Following Cock’s methods to estimate the sample size in pilot trials, 34 participants are needed to produce a one-sided 90% confidence limit of the effect size for the main trial (ie, *d*=0.46) [61]. Assuming the attrition rate of 20% in this pilot trial, we aimed to recruit 34-40 persons in total.

### Randomization

Permuted block randomization with block sizes of 8 people in a ratio of 1:1 was used. A random allocation sequence list was generated using a web-based app, that is, Random.org [62]. An independent research assistant who did not participate in any other parts of the research generated and maintained the random allocation sequence list. This independent research assistant assigned group labels to the participants according to the sequence of their entry, referring to the random allocation sequence list to ensure that the other members of the research team did not foresee the group allocation.

### Statistical Methods

To describe the issue of feasibility (ie, recruitment, retention, participation, and compliance), frequencies and percentages were used. Nonparametric tests were conducted to examine the effects of the intervention. To test the hypothesis on the effects, a Wilcoxon signed-rank test was used to compare the outcome variables before and after the interventions to test the within-group effect in both the groups separately. The level of significance was .05. Missing values were replaced by the last observed values. An intention-to-treat analysis was conducted to interpret the hypotheses [63].

### Ethics

The participants gave their signed informed consent to participate in the study before the study was conducted. Ethical approval was obtained from the Human Subject Ethics subcommittee, The Hong Kong Polytechnic University (Reference Number: HSEARS20180406003).

## Results

### Demographic Data

**Figure 1:**
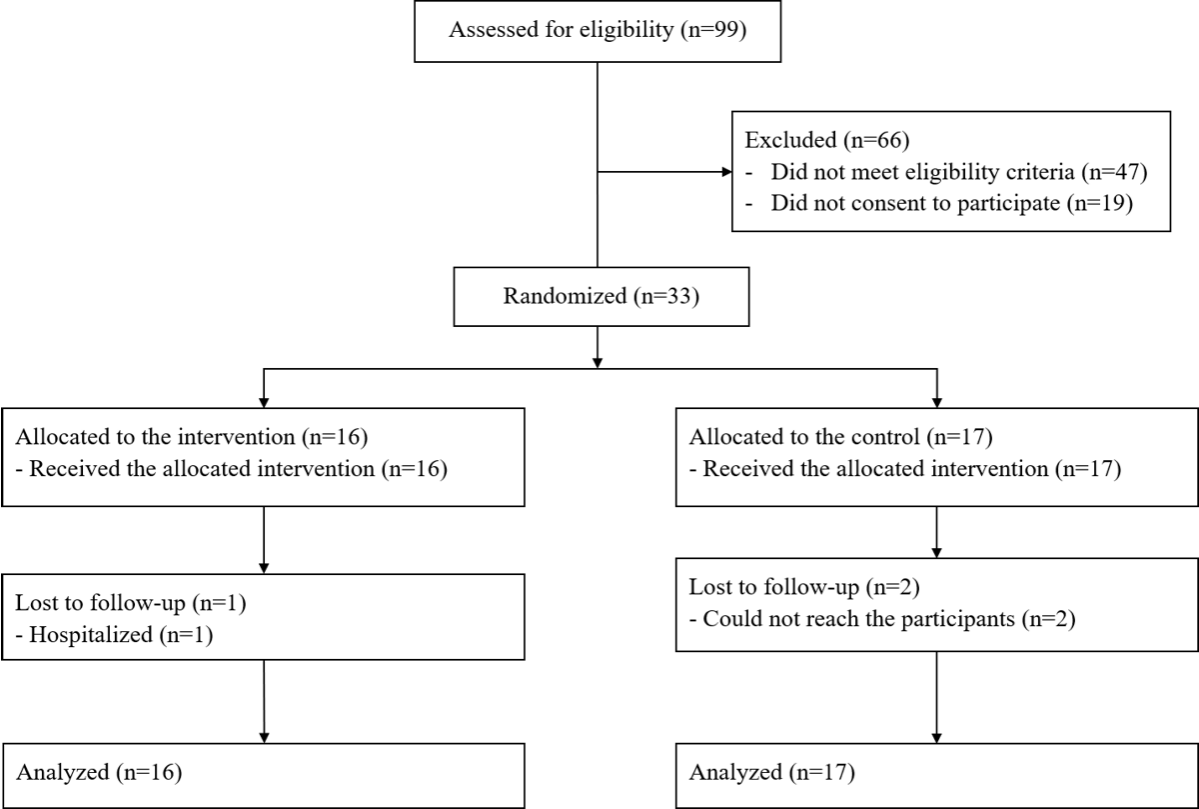
CONSORT flow diagram.

As shown in Figure 1, we screened 99 participants referred to us by the elderly community centers. Sixty-six people were excluded because they did not fulfil the criteria for eligibility or did not give their consent to participate. Thirty-three participants were randomized into either the intervention (n=16) or the control (n=17) group. One participant in the intervention group was lost to follow-up because of hospitalization, and 2 participants in the control group were lost to follow-up because they could not be reached.

As shown in Table 5, the median age of the participants was 71.0 (IQR 9.0) years. The majority of the participants were females (28/33, 85%), had completed secondary school or above (17/33, 52%), and had 1-2 chronic illnesses (17/33, 52%). At the baseline, the median MoCA was 21.0 (IQR 6.5) and the median FFI was 2.0 (IQR 1.5). The median walking time was 149.8 min/day (IQR 77.6). The median step count was 12,256.1 steps/day (IQR 4540.2). The median brisk walking time was 2.6 min/day (IQR 4.8). The 1-min peak cadence was 118.0 steps/min (IQR 20.5). The median MVPA time was 23.0 min/week (IQR 85.0) and 9.0 min/valid day (IQR 22.0). There was no significant difference in all of these variables between the groups at baseline.

**Table 5:** Demographic data and outcomes at baseline.

Demographic data		Total population (N=33)	Intervention group (n=16)	Control group (n=17)	*P* value
**Gender, n (%)**					.58
	Male	5 (15)	3 (19)	2 (12)	
	Female	28 (85)	16 (81)	15 (88)	
Age in years, median (IQR)		71.0 (9)	70.5 (7)	71.0 (14)	.36
**Level of education, n (%)**					.79
	Secondary or above	17 (52)	9 (56)	8 (47)	
	Primary	13 (39)	5 (31)	8 (47)	
	No formal education	3 (9)	2 (12)	1 (6)	
**Chronic illnesses, n (%)**					.90
	0	8 (24)	4 (25)	4 (23)	
	1-2	17 (52)	8 (50)	9 (53)	
	3 or above	8 (24)	4 (25)	4 (23)	
Cognitive function (MoCA^a^), median (IQR)		21.0 (6.0)	21.5 (7.5)	20.0 (5)	.66
Frailty (FFI^b^), median (IQR)		2.0 (1.5)	2.0 (0.8)	2.0 (2.0)	.58
**Physical activity, median (IQR)**					
	Physical activity (PASE^c^)	67.6 (40.3)	62.8 (49.0)	67.6 (31.4)	.18
	Hand-grip strength (kg)	17.3 (2.3)	17.1 (2.3)	17.3 (5.0)	.89
	Walking speed (6MWT^d^)	7.6 (1.7)	7.2 (1.8)	7.7 (2.1)	*.*21
**Walking, median (IQR)**					
	Walking time (min/day)	149.8 (77.6)	158.5 (169.9)	143.4 (64.5)	.14
	Step count (step/day)	12,256.1 (4540.2)	13,057.0 (9644.4)	11,620.7 (2863.5)	.08
	Brisk walking time (min/day)	2.6 (4.8)	3.0 (4.1)	1.0 (4.6)	.11
	1-min peak cadence (step/min)	118.0 (20.5)	122.0 (14.5)	117.0 (24.5)	.34
**MVPA** ^e^ **, median (IQR)**					
	MVPA time (min/week)	23.0 (85.0)	31.5 (226.3)	12.0 (38.5)	.18
	MVPA time (min/valid day)	9.0 (22.0)	8.5 (44.5)	9.0 (14.0)	.38

^a^MoCA: Montreal Cognitive Assessment.

^b^FFI: Fried frailty index.

^c^PASE: Physical Activity Scale for the Elderly.

^d^6MWT: six-minute walk test.

^e^MVPA: moderate-to-vigorous physical activity.

### Objective 1: Feasibility Issues

As shown in Figure 1, the recruitment rate was 33% (33/99). The participant retention rate was 91% (30/33) with 3 participants lost to follow-up. The intervention participation was good. The attendance rate at all the face-to-face training sessions (ie, health education, training in brisk walking, smartphone training, and face-to-face sharing sessions) was 100% (33/33).

As shown in Table 6, in the intervention group (n=16), the mean number of WhatsApp messages received by each participant was 13.5 (SD 6.2) and the mean number of WhatsApp messages sent by each participant was 9.7 (SD 4.5). Most of the messages were read by the participants withing 30 minutes (91/216, 42.1%). The smartphone wearing compliance was good. Almost all of the participants wore the smartphone, as defined by a recording by Samsung Health of at least 1 hour of walking, every day throughout the intervention period with the mean number of wearing days per participant of 54 (SD 1.2) (ie, 97% of the expected wearing days). The reasons for not wearing the smartphone included difficulty in learning how to use it, as stated by 1 participant, who midway through the trial refused to continue wearing the smartphone to implement the intervention, while another participant failed to take the smartphone along on a 2-day trip abroad.

As shown in Table 6, in both groups (N=33), the ActiGraph wearing compliance was very good. The mean number of valid wearing days was 6.9 (SD 0.3) days/week in the intervention group and 6.1 (SD 2.1) days/week in the control group at T0 and 6.4 (SD 0.5) days/week in the intervention and 5.2 (SD 2.7) days/week in the control group at T1. The mean valid wearing time was 875.8 (SD 129.6) min/day in the intervention group and 812.5 (SD 231.9) min/day in the control group at T0 and 901.3 (SD 128.8) min/day in the intervention group and 868.8 (SD 88.8) min/day in the control group at T1. All ActiGraphs were returned with valid data at T0 and the majority of the ActiGraphs in both the groups were returned with valid ActiGraph data (ie, adequate valid wearing minutes and days for analysis) at T1.

**Table 6:** Smartphone and ActiGraph use and compliance.

Use of smartphones/ActiGraph		Intervention group, (n=16)	Control group, (n=17)
**WhatsApp messages in the whole intervention period (10 weeks), mean (SD)**			
	Messages received by each participant	13.5 (6.2)	N/A^a^
	Messages sent by each participant	9.7 (4.5)	N/A
**WhatsApp message reading time (total messages=216), n (%)**			
	<30 min	91 (42.1)	N/A
	30 min-1 h	35 (16.2)	N/A
	1-2 h	17 (7.9)	N/A
	2-24 h	36 (16.7)	N/A
	Unread	37 (17.1)	N/A
**Smartphone compliance, mean (SD)**			
	Valid wearing days per participant (range 0-56 days)	54.1 (1.2)	N/A
**ActiGraph compliance at T0^b^** **, mean (SD)**			
	Valid wearing day per participant (range 0-7 days)	6.9 (0.3)	6.1 (2.1)
	Valid wearing minute per participant per day (range 0-1440 min)	875.8 (129.6)	812.5 (231.9)
**ActiGraph compliance at T1^c^** **, mean (SD)**			
	Valid wearing day per participant (range 0-7 days)	6.4 (0.5)	5.2 (2.7)
	Valid wearing minute per participant per day (range 0-1440 min)	901.3 (128.8)	868.8 (88.8)
**Returns with valid ActiGraph data at T0, n (%)**			
	Valid return	16 (100)	17 (100)
	Invalid return	0	0
	Unworn	0	0
**Returns with valid ActiGraph data at T1, n (%)**			
	Valid return	13 (81)	12 (71)
	Invalid return	2 (13)	3 (18)
	Unworn	1 (6)	2 (12)

^a^Not applicable.

^b^T0: beginning of the intervention.

^c^T1: one week after the intervention.

### Objective 2: Effects

As shown in Table 7, after the interventions, cognitive function significantly improved in both the intervention (median difference 2.5, *P*=.003) and control (median difference 1.0, *P*=.009) groups. There was a significant reduction in frailty after the intervention in the intervention group (FFI median difference –1.0, *P*=.005). Walking time (median difference 57.9 min/day, *P*=.03), step count (median difference 3778.9, *P*=.02), brisk walking time (median difference 3.1 min/day, *P*=.009), and peak cadence (median difference 7.0 steps/min, *P*=.003) increased significantly after the intervention in the intervention group only but not in the control group. MVPA time (median difference 86 min/week, *P*=.04; median difference 18.5 min/valid day, *P*=.02) increased significantly after the intervention in the intervention group only. The effect sizes of all the outcomes (ie, Cohen *d* between T0 and T1) in the intervention group were higher than those in the control group.

**Table 7:** Within-group effects of the interventions before and after the interventions in each group.

Outcome variables		Intervention group (n=16)				Control group (n=17)				
		T0^a^	T1^b^	*P* value^c^	ES^d^		T0	T1	*P* value	ES
Cognitive function (MoCA^e^), median (IQR)		21.5 (7.5)	24.0 (6.5)	.003	0.70		20.0 (5.5)	21.0 (6.0)	.009	0.35
Frailty (FFI^f^), median (IQR)		2.0 (1.0)	1.0 (0.8)	.007	–1.41		2.0 (2.0)	1.0 (2.0)	.06	–0.29
**Physical activity, median (IQR)**										
	Physical activity (PASE^g^),	62.8 (49.0)	99.4 (46.6)	.002	1.13		67.6 (31.4)	77.4 (35.4)	.43	0.21
	Hand-grip strength (kg)	17.0 (2.2)	18.7 (5.7)	.009	0.66		17.3 (5.0)	16.8 (5.5)	.41	0.12
	Walking speed (6MWT^h^)	7.2 (1.8)	5.5 (1.2)	.001	–1.32		7.7 (2.1)	7.3 (1.7)	.23	–0.33
**Walking,** **median (IQR)**										
	Walking time (min/day)	158.4 (169.9)	216.3 (106.9)	.03	0.23		143.4 (64.5)	141.3 (126.6)	.91	–0.05
	Step count (step/day)	13,057.0 (9644.4)	16,835.9 (7019.3)	.02	0.39		11,620.7 (2863.5)	10,935.3 (7174.9)	.80	0.03
	Brisk walking time (min/day)	3.0 (4.1)	6.1 (6.7)	.009	0.58		1.0 (4.6)	1.0 (8.0)	.26	0.34
	1-min peak cadence (step/min)	122.0 (14.5)	129.0 (27.3)	.003	0.72		117.0 (24.5)	118.0 (14.5)	.28	0.21
**MVPA**										
	MVPA^i^ time (min/week)	31.5 (226.3)	117.5 (294.5)	.04	0.35		12.0 (38.5)	14.0 (28.0)	.07	–0.43
	MVPA time (min/valid day)	8.5 (44.5)	27.0 (37.8)	.048	0.49		9.0 (14.0)	9.0 (20.0)	.08	0.27

^a^T0: beginning of the intervention.

^b^T1: one week after the intervention.

^c^Results based on Wilcoxon signed-rank test.

^d^ES: effect size (Cohen *d* between T0 and T1).

^e^MoCA: Montreal Cognitive Assessment.

^f^FFI: Fried frailty index.

^g^PASE: Physical Activity Scale for the Elderly.

^h^6MWT: six-minute walk test.

^i^MVPA: moderate-to-vigorous physical activity.

## Discussion

To summarize the results, this is the first study to provide direct evidence to show that mHealth interventions are not only effective at changing the behavior of young people or healthy older people but also at increasing the walking and MVPA time of older people with cognitive frailty. The extent of the increase through this mHealth intervention was enough to lead to a significant reduction in frailty in the intervention group but not in the control group. The extent of the increase in the MVPA time led to an increase in the cognitive function in both the groups, but the effect size was larger in the intervention group. Our findings confirm that the implementation of mHealth interventions by using smartphones and ActiGraphs is highly feasible, with good recruitment, compliance, and retention.

The participants in our study in Hong Kong walked (12,256.1 steps/day) much more than those in a study conducted in the United States (5660.8 steps/day) [64]. The reason for this is that the pedestrian infrastructure in Hong Kong is good, and people in Hong Kong spend a lot of time walking from one place to another, as reported in a previous study (569 min/week) [65]. This supports the idea that using brisk walking as the target exercise in pedestrian-friendly societies is highly feasible. Although their walking time is relative long, the brisk walking and MVPA times of older people with cognitive frailty are still suboptimal.

Recently, it has been proposed that cognitive frailty can potentially be reversed [5,66,67]. However, there is a lack of direct evidence to show that interventions are effective at reversing cognitive frailty. This study provides direct evidence that cognitive frailty is reversible by increasing MVPA. Further studies should examine whether this reversal of cognitive frailty can be sustained by engagement in physical activities and whether it will eventually lead to a reduced risk of dementia and onset of dependence.

Longitudinal observational studies have shown that there is a significant association between high levels of physical activity and reduced risks of cognitive decline in older people [68]. A systematic review showed that there are only a limited number of trials supporting the claim that physical activity improves the cognitive function in older people with MCI [69]. However, in these studies, physical vulnerability (ie, frailty) was not taken into account when selecting participants with MCI. There is evidence that the majority of people with MCI have a coexisting condition of frailty (ie, cognitive frailty), which is associated with adverse health outcomes (eg, malnutrition, depression) to a significantly larger extent than solely having MCI [70]. This shows that people with MCI without frailty may not be the group to target to benefit from physical activity nor is their condition necessarily associated with adverse health outcomes. This study provides new knowledge that physical activity training for people with MCI plus concurrent frailty (ie, cognitive frailty) can improve their cognitive functions. Further studies should examine whether increased physical activity can lead to a reduction in adverse health outcomes (eg, malnutrition, depression).

Recent systematic reviews have shown that eHealth interventions are effective at encouraging older people (age >50 years) to engage in physical activity [25]. In these studies, there is no evidence to show that eHealth is also effective among vulnerable older people (ie, older people with cognitive frailty). Fatigue (also known as exhaustion) is also a phenotypic component of frailty [2]. Fatigue contributes to reduced physical activity in older people, making it more difficult to implement an intervention designed to enhance their engagement in physical activity [71]. As mentioned earlier, the most frequently cited barriers to participation in physical activity include a lack of company, a lack of interest, a lack of opportunities to engage in sports, and a lack of transportation [20]. An attempt was made in this study to develop an intervention by solving these problems through integrating eHealth technology with training in brisk walking in daily-living venues. For example, we began with individualized starting doses and targets to reduce the difficulties of participation for people with frailty (ie, tailored dosages), formed groups on an e-platform to enhance social support (ie, WhatsApp), used a physical activity–tracking app to enhance interest (ie, Samsung Health), provided an opportunity-free form of sports (ie, brisk walking), and offered a transportation-free form of exercise (ie, walking in public parks close to the participants’ homes). This study provides proof that the eHealth intervention resolves the abovementioned barriers to engaging in physical activity faced by vulnerable older people with cognitive frailty and creates a more favorable environment for them. Therefore, these reasons show the feasibility of implementing this intervention. This intervention is also effective at increasing the MVPA of older people, to an extent that is sufficient to lead to clinical improvements in their cognitive function and physical frailty. A longer period of engagement in increased physical activity can even lead to neurological changes in older people with MCI (eg, increase hippocampal volume) [72]. Further studies should be conducted to examine the long-term effect of eHealth interventions on MVPA as well as on beneficial neurological changes.

Apart from resolving the barriers faced by the participants, the eHealth intervention in our study provided more e-contacts to the participants. A systematic review showed that remote feedback (eg, e-contact) is at least equally effective as supervised training and is more effective than usual care or no treatment, and more contacts are associated with better compliance with physical activity in older people [73]. This intervention employed mHealth technologies to strengthen multiple behavioral change techniques such as e-contacts and social support. mHealth interventions probably allow more frequent, higher quality, and more cost-effective contacts compared with conventional behavior change interventions (eg, face-to-face or telephone-based interventions). With the advancement of smart technology, nonhuman conversational agents powered by artificial intelligence (eg, Chatbot) can also play the role of e-coaching and connect with e-contacts to promote people’s health [74]. Further studies should examine the components (eg, social support, contact) that are more effective in yielding clinical benefits and those components should be strengthened to achieve these benefits. Further, mHealth interventions in the future should be upgraded with new technologies.

Older people, particularly those with chronic conditions, find it difficult to meet MVPA exercise targets (ie, >150 min/week) [54]. This study showed that even if the participants could not achieve the median MVPA performance of 150 min/week during the intervention, after the intervention, beneficial clinical effects on cognitive frailty were also observed. This study recommends implementing an eHealth intervention targeted at increasing the MVPA time of older people with cognitive frailty to an extent that they can tolerate, even for those who are unable to attain an MVPA level of >150 min/week.

There are several limitations to this study. First, a relatively small sample of participants was recruited, which may limit the generalizability. The effect size with a better confidence should be confirmed by a large-scale RCT. Second, the data collectors were not blinded; therefore, the results may be affected by observation bias. Third, the participants in this study had a high level of step count (median 12,256.1 steps/day); therefore, this study has limited generalizability to people who do not walk often. Further, participants in the intervention had slightly more active walking behaviors because of random variation in a small sample, particularly an almost significantly higher level of step count (*P*=.08). The participants in the intervention group may be more motivated to start with the intervention. Fourth, significant improvement in the cognitive function was also observed in the control group without significant improvement in the physical activity. This may probably be caused by the practice effects of the MoCA [75]. The actual cognition-enhancing effect of mHealth intervention should be interpreted with caution. Fifth, in the mHealth group, the participants unavoidably received more e-contacts than the control group. The number of contacts may possibly be a confounding factor. These factors should be controlled in the main trials. Finally, this study did not measure the engagement (eg, contacts or social interactions) in both the groups. This study could not conclude whether the possibly increased engagement is caused by the mHealth measures.

In conclusion, it is feasible to implement this mHealth intervention in older people with cognitive impairment. The mHealth intervention is effective at enhancing compliance with the brisk walking training program delivered by conventional behavior change interventions. This study provides preliminary evidence that this mHealth intervention can increase MVPA time to an extent that is sufficient to yield clinical benefits (ie, a reduction in cognitive frailty). Future studies should employ a full-powered and assessor-blinded RCT to warrant these effects.

## Appendix

Multimedia Appendix 1 CONSORT-eHEALTH (V 1.6.1): Submission/Publication Form.

Multimedia Appendix 2 Intervention tailoring strategies on setting personalized goals and contacting subjects.

## Abbreviations

FFI: Fried frailty index
MCI: mild cognitive impairment
mHealth: mobile health
MoCA: Montreal Cognitive Assessment
MVPA: moderate-to-vigorous physical activity
RCT: randomized controlled trial
WHO: World Health Organization
