# Supplementary material for: Effects of an mHealth Brisk Walking Intervention on Increasing Physical Activity in Older People With Cognitive Frailty: Pilot Randomized Controlled Trial
Source: JMIR Mhealth Uhealth. 2020 Jul 31;8(7):e16596. doi: 10.2196/16596 (PMC7428907; doi:10.2196/16596)
Supplement: Multimedia Appendix 2 [file mhealth_v8i7e16596_app2.docx]

Appendix 1: Intervention tailoring strategies on setting personalized goals and contacting subjects

1. ***Setting personalized goals***: The exercise goals were set according to four principles: a) practice availability, b) baseline fitness, c) previous performance, and d) personal wish. At baseline, we discussed with the subjects to find out time slots that they are available to practice (e.g., morning exercise, after daily grocery shopping). Then, we set the number of time slots and periods to practice brisk walking that the participants were available. According to the baseline fitness of the subjects, we started the goal of exercise individually. Pre-frail older people started with a higher goal of 5-10 sessions of brisk-walking per week. A starting session is a continuous brisk walking for 10 minutes. Frail older people started with a lower goal of 3-5 sessions of brisk-walking per week. Goals were revised weekly. In the subsequent weeks, the exercise goals were set according to the performance of the previous weeks. For those who could achieve the weekly goal (i.e., the achievers), 2-5 minutes were added on each session of brisk walking and one more session was added. For those who could not achieve (i.e., non-achiever), the goal remained as the best performance the subject has ever achieved in the previous weeks. Finally, the final goal was compromised with the subjects according to their wish (e.g., confidence and motivation) through WhatsApp messaging.
2. ***Contacting subjects:*** WhatsApp messages were sent to subjects in response to three triggers: a) weekly routine messages, b) when there is no brisk walking observed for more than 2 days, and c) when the weekly goal is achieved before the end of the week. Every week, a set of standardized pre-scripted health benefits of brisk walking was set to the subjects. Also, a summary of subjects’ performance in the previous week were sent to the subjects. The interventionist filled in a template with subjects’ performance related figures to form summary. When there was no brisk walking observed for more than two days, e-reminders (e.g., “you have not practiced brisk walking”) and e-coaching (e.g., providing training guidance on walking place, walking duration, walking speed, steps on using Samsung Health) were sent by the interventionist. E-reminders were selected from a list of pre-scripted messages. E-coachings were individualized aiming to provide solution and guidance on participants’ difficulties. When the weekly goal was achieved before the last day of the week, messages of praise was selected from a bank (e.g., “I know you can do it”, “Carry on”) were sent by the interventions to the subjects. At the moment when the goal was achieved, messages of praise and e-tokens (e.g., a trophy sticker) were also automatically sent by the Samsung Health.
